# Current Trends and Future Directions in Lumbar Spine Surgery: A Review of Emerging Techniques and Evolving Management Paradigms

**DOI:** 10.3390/jcm14103390

**Published:** 2025-05-13

**Authors:** Gianluca Galieri, Vittorio Orlando, Roberto Altieri, Manlio Barbarisi, Alessandro Olivi, Giovanni Sabatino, Giuseppe La Rocca

**Affiliations:** 1 Institute of Neurosurgery, Fondazione Policlinico Universitario A. Gemelli IRCCS, Catholic University, 00168 Rome, Italy; vittorio.orlando01@icatt.it (V.O.); alessandro.olivi@policlinicogemelli.it (A.O.); giovanni.sabatino@policlinicogemelli.it (G.S.); giuseppe.larocca@policlinicogemelli.it (G.L.R.); 2 Neurosurgical Training Center and Brain Research, Mater Olbia Hospital, 07026 Olbia, Italy; 3 Multidisciplinary Department of Medical-Surgical and Dental Specialties, University of Campania “Luigi Vanvitelli”, 81100 Caserta, Italy; roberto.altieri@unicampania.it (R.A.); manlio.barbarisi@unicampania.it (M.B.)

**Keywords:** lumbar spine surgery, minimally invasive techniques, robotic-assisted surgery, augmented reality navigation, artificial intelligence

## Abstract

**Background/Objectives**: Lumbar spine surgery has undergone significant technological transformation in recent years, driven by the goals of minimizing invasiveness, improving precision, and enhancing clinical outcomes. Emerging tools—including robotics, augmented reality, computer-assisted navigation, and artificial intelligence—have complemented the evolution of minimally invasive surgical (MIS) approaches, such as endoscopic and lateral interbody fusions. **Methods**: This systematic review evaluates the literature from February 2020 to February 2025 on technological and procedural innovations in LSS. Eligible studies focused on degenerative lumbar pathologies, advanced surgical technologies, and reported clinical or perioperative outcomes. Randomized controlled trials, comparative studies, meta-analyses, and large case series were included. **Results**: A total of 32 studies met the inclusion criteria. Robotic-assisted surgery demonstrated high accuracy in pedicle screw placement (~92–94%) and reduced intraoperative blood loss and radiation exposure, although long-term clinical outcomes were comparable to conventional techniques. Intraoperative navigation improved instrumentation precision, while AR enhanced ergonomic workflow and reduced surgeon distraction. AI tools showed promise in surgical planning, guidance, and outcome prediction but lacked definitive evidence of clinical superiority. MIS techniques—including endoscopic discectomy and MIS-TLIF—offered reduced blood loss, shorter hospital stays, and faster recovery, with equivalent pain relief, fusion rates, and complication profiles compared to open procedures. Lateral and oblique approaches (XLIF/OLIF) further optimized alignment and indirect decompression, with favorable perioperative metrics. **Conclusions**: Recent innovations in lumbar spine surgery have enhanced technical precision and perioperative efficiency without compromising patient outcomes. While short-term benefits are clear, long-term clinical advantages and cost-effectiveness require further investigation. Integration of robotics, navigation, AI, and MIS into spine surgery reflects an ongoing shift toward personalized, data-driven, and less invasive care.

## 1. Introduction

Lumbar spine surgery (LSS) has evolved dramatically over the past several decades, driven by a desire to improve patient outcomes and reduce the morbidity associated with traditional open procedures. Early spinal surgeries in the 20th century, such as the first lumbar discectomy in 1908 and spinal fusions reported in 1911, established foundational techniques [1,2]. In the last decades, conventional open approaches were flack by the adoption of minimally invasive techniques and novel technologies in lumbar surgery. The rationale for embracing new methods is multifold: to minimize tissue trauma and postoperative pain, enhance the accuracy of implant placement, reduce complications such as neurovascular injuries from misplaced hardware, and improve overall surgical precision and consistency. For example, computer-assisted navigation and robotic guidance were introduced to reduce screw misplacement rate and boost accuracy. Likewise, the need to reduce surgeons’ radiation exposure during fluoroscopy and to streamline operative workflow has led to innovations like intraoperative 3D navigation, augmented reality (AR) displays, and robotic platforms. Patient radiation exposure is an equally important concern: traditional fluoroscopy-guided procedures, particularly when extensive imaging is needed for instrument placement, can result in significant cumulative radiation doses to the patient and surgeons. Innovations have been shown to reduce the number of repeated fluoroscopy shots; nevertheless, technologies like intraoperative CT scans or 3D imaging systems may deliver higher single-dose exposures compared to standard fluoroscopy, highlighting the need for careful radiation management protocols.

In recent years, artificial intelligence (AI) has also emerged as a cutting-edge tool, with potential applications in surgical planning, imaging analysis, and intraoperative guidance. Overall, the past five years have seen a rapid uptake of these technologies alongside refined minimally invasive surgical (MIS) techniques (endoscopic and percutaneous approaches), signaling a paradigm shift in how lumbar spine surgery is performed.

This review provides a systematic and critical appraisal of the latest advancements in lumbar spine surgery, focusing on peer-reviewed evidence from the last five years. Key areas include technological innovations (robotics, AR, navigation, and AI), MIS techniques (endoscopic and lateral approaches), comparisons of these innovations to conventional surgery, and their impact on clinical and functional outcomes.

## 2. Materials and Methods

### 2.1. Study Design and Search Strategy

This review was conducted following a systematic approach to identify, appraise, and synthesize recent advancements in LSS. We performed a comprehensive literature search in February 2025 across multiple databases, including PubMed/Medline, Research Gate, Google Scholar, and Cochrane, focusing on publications published between February 2019 and February 2025. The following search terms were used: “lumbar spine surgery”, “machine learning”, “artificial intelligence”, “robotic”, “endoscopic”, and “minimally invasive”. Additional studies were identified through manual reference screening of relevant reviews and meta-analyses.

### 2.2. Inclusion Criteria

Studies were included if they met the following criteria:

- Published in a peer-reviewed journal indexed in PubMed from February 2020 onward.
- Focused on lumbar spine surgery, addressing degenerative conditions, deformity correction, or other lumbar pathologies.
- Utilized advanced technologies, including robotic-assisted surgery, computer navigation, AR, AI, or MIS techniques such as full-endoscopic discectomy, percutaneous instrumentation, and lateral/oblique lumbar interbody fusion (XLIF/OLIF).
- Reported relevant clinical and surgical outcomes, such as accuracy of instrumentation, perioperative metrics (operative time, blood loss, and hospital stay), complication rates, and functional outcomes (pain scores, disability indices, and fusion rates).
- Study types: randomized controlled trials (RCTs), prospective or retrospective comparative studies, systematic reviews, meta-analyses, and large case series were included if higher-level evidence was lacking (e.g., for AR or AI applications where RCT data remain sparse).

### 2.3. Exclusion Criteria

We excluded studies that included the following:

- Were case reports or small case series without comparative data.
- Were conference abstracts or non-peer-reviewed sources.
- Focused on cervical or thoracic spine surgery, unless findings were generalizable to lumbar procedures.
- Were review papers that did not provide new data (these were used for background information only).
- Contained overlapping patient cohorts—in such cases, the most comprehensive or recent study was selected to avoid duplication.
- Non-English-language publications.

### 2.4. Study Selection and Data Extraction

All identified titles and abstracts were screened by two independent reviewers for relevance. Full-text articles of potentially eligible studies were retrieved and assessed against the inclusion criteria. Disagreements were resolved by consensus or consultation with a third reviewer. A PRISMA flow diagram was used to document the study selection process.

Data extracted from the included studies included the following: study design (RCT, cohort, and meta-analysis); patient population (sample size, demographics, and pathology treated); surgical technique/technology evaluated; key outcomes (accuracy, operative time, blood loss, hospital stay, complications, pain/disability scores, and fusion rates); quality assessment of evidence (RCT risk of bias, assessing the methodological of systematic reviews (AMSTAR) score for systematic reviews when available).

Due to study heterogeneity, a formal meta-analysis was not performed, and findings were summarized narratively.

## 3. Results

**Figure 1:**
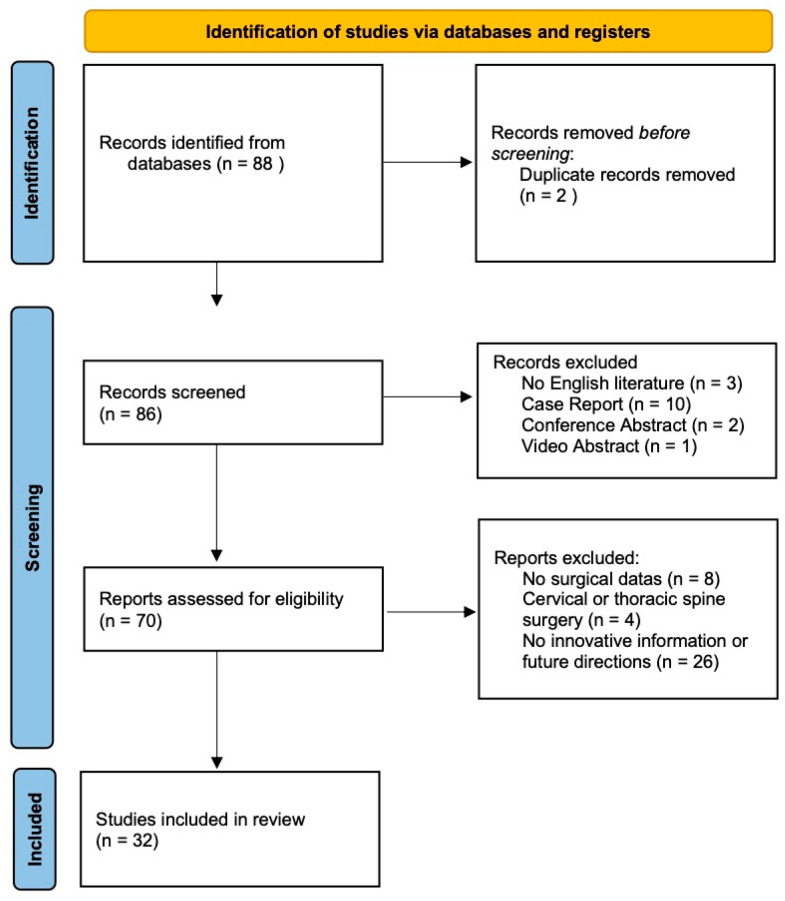
PRISMA 2020 flow diagram for new systematic reviews which included searches of databases and registers.

The screening process is illustrated in Figure 1. A total of 88 studies were initially identified through database searches. Following title and abstract screening, 56 publications were excluded for the following reasons: 2 duplicates, 3 non-English language articles, 10 case reports, 4 studies focused on cervical or thoracic spine surgery, 8 articles presenting non-surgical data, 26 lacking innovative content or future perspectives, 2 conference abstracts, and 1 video article. Ultimately, 32 studies met the inclusion criteria and were included in this review.

### 3.1. Technological Innovations in Lumbar Spine Surgery

#### 3.1.1. Robotic-Assisted Surgery

Robotic-assisted spine surgery has seen increasing adoption in the past five years, particularly for pedicle screw placement and lumbar fusion procedures. The primary demonstrated benefit of robotic systems is improved accuracy and consistency of instrumentation [3]. Conventional freehand pedicle screw placement relies on the surgeon’s anatomical knowledge and fluoroscopic guidance; in inexperienced hands, misplacement rates as high as ~30% in the lumbar spine and 55% in thoracic spine have been reported [2]. In contrast, modern robotic guidance platforms, which use preoperative or intraoperative imaging to plan screw trajectories and guide tools, achieve placement accuracy in the range of ~92–94% [2,4]. Multiple recent studies and meta-analyses confirm that robotics significantly increases the proportion of screws placed in the optimal position compared to conventional techniques [5]. This precision is especially valuable in MIS approaches where anatomic landmarks are not directly visible [2].

A 2023 updated systematic review of 20 RCTs and a total of 954 patients compared robot-assisted versus conventional spine surgery. The meta-analysis found that robot-assisted surgery was associated with less intraoperative blood loss and lower intraoperative radiation dose exposure compared to freehand techniques [2]. Hospital length of stay was also modestly shorter in the robotic groups on average [2,5]. These benefits likely stem from the robot’s ability to facilitate minimally invasive screw placement (smaller incisions and less muscle dissection) and more efficient, planned trajectories (reducing the need for repeated fluoroscopy shots) [6,7]. However, one trade-off can be increased operative setup time or technical troubleshooting, although the RCT meta-analysis found no significant difference in total operative duration between robotic and freehand cohorts [2]. Early iterations of robotic systems did face learning-curve challenges, but as surgeons become more experienced, operative times have become comparable to non-robotic procedures [3].

Despite clear gains in screw accuracy and perioperative parameters, improvements in patient-centered clinical outcomes with robotics have not yet been conclusively demonstrated. The meta-analyses of RCTs reported no significant differences in postoperative low back pain scores or disability indices between robotic and conventional surgery patients [2]. At early follow-up, patients experienced similar pain relief and functional improvement regardless of whether a robot was used for instrumentation. Similarly, rates of overall complications (infection, neurological injury, etc.) did not differ appreciably between groups in most studies [2,3]. A large multicenter database study (2015–2022) comparing robotic-assisted vs. navigation-assisted lumbar fusions found no reduction in 90-day adverse events with robotics; the robotic cohort had a slightly higher readmission rate, possibly reflecting early adoption hurdles, and no difference in 3-year reoperation rates [8]. These findings underscore that, while robots enhance technical execution, the incremental advantage in clinical efficacy remains unproven [2]. Table 1 summarizes key outcomes of robotic-assisted versus conventional approaches.

Several studies have confirmed that robotic-assisted spine surgery maintains low complication rates. In a multicenter study of 799 robotic procedures with 4838 pedicle screws, the intraoperative complication rate was reported at 3.13%, and the 90-day implant-related revision rate was 0.88% [9]. Good et al. (2021) found that 97% of robotic-guided patients remained complication-free at 90 days versus 73% with fluoroscopy guidance [10].

The value proposition of robotics is still being evaluated. Robots entail substantial capital and maintenance costs, and longer-term studies on cost-effectiveness are only beginning to emerge. One recent single-center analysis suggested that robotic spinal surgery can become cost-effective in the context of reducing revision surgeries, preventing complications like misplaced screws that would require reoperation [3,11]. In that model, the incremental cost-effectiveness ratio improved markedly for cases requiring instrumentation of only 1–2 levels, and the authors concluded that if robotic use averts even a few costly complications, it may justify the investment [11]. Nonetheless, high-quality evidence on cost-benefit is scarce: the 2023 RCT meta-review noted that studies of cost-effectiveness are still very rare [2]. Hospitals must weigh the upfront expense against potential gains in efficiency and avoidance of complications. As robotic technology matures and competition increases, costs may decrease, further influencing adoption.

Overall, robotic systems offer greater surgical precision, but the lack of superior clinical outcomes thus far suggests that accuracy alone is not enough to improve metrics like pain or fusion rate in the short term, or that existing conventional techniques were already performing adequately in those domains [3]. It may be that the true benefits of robotics will manifest in specific scenarios (like complex deformity cases or reducing radiation to operating room staff) or over longer follow-up (as lower rates of screw-related complications over years). Continued research, including randomized trials focusing on patient outcomes and cost, is ongoing to determine the precise role of robotics in lumbar spine surgery.

#### 3.1.2. Intraoperative Navigation and Augmented Reality

Intraoperative navigation systems, non-robotic computer-assisted, have been used in spine surgery for over two decades and represent a stepping stone between freehand techniques and robotic automation [12,13]. Standard navigation typically involves obtaining 3D imaging, such as intraoperative Computer Tomography (CT) or O-arm scans and using optical trackers to guide instrument placement on a virtual model of the patient’s spine [14]. Numerous studies have shown that navigation improves pedicle screw placement accuracy compared to freehand methods, increasing the proportion of correctly placed screws and thereby potentially reducing neural impingement or breach of cortical bone [15,16]. Navigation is especially valuable for anatomically challenging cases or deformities where landmarks are distorted. However, traditional navigation systems have drawbacks: surgeons must shift their gaze between the surgical field and a remote monitor, and the setup and registration process can be time-consuming, which disrupts operative room (OR) workflow [13,15].

Recent evidence suggests that robotic systems may offer slightly greater precision and workflow efficiency in specific contexts. For instance, Heath et al. found that robot-assisted TLIF procedures achieved higher clinical accuracy than navigation-guided techniques (100% vs. 92.1% acceptable screw placement, *p* < 0.001), although total operative time was longer in two-level procedures [17]. Similarly, Sundaram et al. reported that robotic assistance in OLIF reduced operative times by up to 62 min and improved surgical efficiency [18]. Additionally, Shahi et al. demonstrated that robotic guidance was associated with significantly lower radiation exposure to both surgeons and patients when compared to navigation [19]. These findings suggest that, while both technologies enhance surgical precision, robotics may confer additional benefits in selected patient populations or complex procedures. Nevertheless, the added complexity, resource availability, and steep learning curves associated with both robotic and navigation platforms have historically limited their widespread adoption in spine surgery, as some surgeons reported longer operative times and technical integration challenges [15]. One of the most significant barriers to widespread adoption is the economic cost. These systems often require substantial upfront investment for the acquisition of hardware (e.g., O-arm, navigation towers, and reference arrays) and software licenses. There are ongoing maintenance and calibration expenses, as well as training costs for staff and surgeons. A 2022 cost-analysis study reported that the use of intraoperative navigation in lumbar fusion procedures increased operative costs by up to $2500–$3000 USD per case, mainly due to the imaging equipment and disposables required [20]. These limitations have spurred development of newer solutions like AR [12,13].

Augmented reality technology seeks to address the workflow issues of traditional navigation by superimposing digital navigation cues directly into the surgeon’s field of view. In AR-assisted spine surgery, the surgeon can see the patient’s anatomy or a holographic trajectory for instrumentation, overlaid on the actual operative field, either via a head-mounted display or an optical projection system [21]. This allows the surgeon to keep their eyes on the patient while still visualizing critical internal anatomy or instrument paths [22]. A 2021 systematic review of AR navigation in spine surgery included 28 studies and found that AR systems provided superior workflow and non-inferior accuracy compared to conventional navigation or freehand techniques [15]. Essentially, AR can maintain the accuracy benefits of computer guidance while improving ease of use; surgeons reported a smoother workflow and less distraction using AR, as they did not need to look away to a separate screen [15]. Early evidence also indicates AR may reduce radiation exposure: by relying on pre-acquired imaging and continuous visual guidance, AR navigation can decrease the need for repeated fluoroscopy shots, although data on radiation were limited and mixed in the review [15,21,22].

Critically, while AR-assisted surgery appears technologically promising, the evidence base is still nascent. The systematic review noted that there were no studies yet reporting on long-term clinical outcomes, complication rates, or cost-benefit for AR in spine surgery [15,22]. Most included studies were feasibility studies, case series, or small comparative trials focusing on technical metrics like screw accuracy and workflow [23]. So far, AR has been shown to achieve pedicle screw accuracy on par with standard navigation and clearly better than unguided freehand [15]. For example, one study in the review found head-mounted display AR was comparable in accuracy to conventional navigation, though another noted that some AR systems still had kinks to work out [15]. Overall, no significant accuracy trade-offs have been observed with AR, meaning surgeons can adopt AR without losing precision. The main advantage lies in ergonomics and efficiency: AR offers a more intuitive interface that may shorten the learning curve for navigation and encourage more surgeons to use guidance technology. Indeed, AR is viewed as an important innovation to promote wider adoption of computer-assisted spine surgery by overcoming the distraction factor of older systems [15,22].

Table 1 provides an overview of AR versus conventional navigation.

#### 3.1.3. Artificial Intelligence Applications

Artificial intelligence in spine surgery is an emerging frontier that overlaps with many of the technologies above. Broadly, AI refers to computer algorithms, including machine learning and deep learning models, that can analyze complex data, recognize patterns, and assist in decision-making. In the context of lumbar spine surgery, AI has been explored in several domains [24].

AI algorithms have been developed to interpret spinal imaging to detect pathologies and even perform automated measurements [25]. They can generate 3D reconstructions of the spine and help plan optimal surgical trajectories [26]. This can assist surgeons in preoperative planning by simulating surgery and identifying potential challenges before making an incision [27].

AI is being integrated with robotic surgery systems to enhance their capabilities. For example, machine learning models can optimize the robotic trajectory planning for pedicle screws or adjust in real-time to anatomic variations, potentially increasing the dexterity and autonomy of robotic assistants [26]. Some robotic platforms incorporate AI for image recognition and instrument tracking. Additionally, AI-driven analytics can process the navigation data to provide the surgeon with real-time feedback or alerts: for example, warning if a planned screw is too close to a nerve root [24,28].

AR offers a visual overlay; pairing it with AI could enable advanced features like automatic anatomical labeling in the surgeon’s view or highlighting of critical structures. Recent reviews emphasize that the incorporation of AI with AR/VR is promising, with potential to further enhance surgeon performance and safety [26]. For instance, an AI might analyze the live endoscopic feed during an AR-assisted procedure to identify tissue types or flag abnormal anatomy, complementing the AR visualization [26].

AI can sift through large patient datasets to predict outcomes of lumbar surgery [29]. Models have been created to forecast which patients will improve after a fusion or which are at risk of complications, based on preoperative variables (imaging, demographic, and health factors) [26]. Such predictive tools could someday guide patient selection or personalized surgical approaches: for example, AI might suggest that a certain patient would benefit more from an MIS approach vs. open or predict the likelihood of fusion success with or without adjuncts [25,27,29].

AI in spine surgery is in early stages of clinical application. A 2024 systematic review on AI-assisted spine surgery identified only 11 relevant studies in the last decade, reflecting how nascent this field is [26]. The review concluded that no current evidence shows superiority of AI-assisted techniques over conventional surgery in terms of patient outcomes [26]. In other words, while various AI tools have been tested in pilot studies or retrospective analyses, none have yet proven that using AI leads to better pain relief, higher fusion rates, or other tangible patient benefits. However, those studies highlight that AI-assisted systems are generally feasible and safe, and they can improve certain process measures, can reduce dependence on fluoroscopy, and maintain or improve accuracy of screw placement relative to standard fluoroscopic guidance [26].

One concrete example is the use of AI in the automated planning of pedicle screw trajectories: algorithms can recommend the optimal entry point and angle based on each patient’s CT scan, which the surgeon can then follow with or without a robot [28]. Studies show this can be highly accurate and may shorten planning time [30]. Another area is AI-driven monitoring: systems that analyze the sound or resistance feedback when a pedicle is being probed, to warn if the bone is thinning. These kinds of “smart” tools are under development [15].

AI has also been applied to postoperative outcomes; for example, machine learning models have been used to predict which patients will achieve a certain percentage of Oswestry Disability Index (ODI) improvement at 1 year after lumbar fusion or to classify MRI (Magnetic Resonance Imaging) images to decide if a patient is a surgical candidate. In one imaging study, an AI-assisted interpretation of lumbar MRI had a sensitivity of ~94.5% for detecting certain degenerative changes, slightly outperforming human readings [27,29,31,32].

Beyond digital technologies, emerging areas such as regenerative medicine and nanotechnology are opening new frontiers in lumbar spine surgery. Regenerative strategies, including stem cell therapy and biologics, aim to restore intervertebral disc health or enhance bony fusion. Mesenchymal stem cells (MSCs), derived from sources like bone marrow, adipose tissue, and umbilical cord, have shown promise in preclinical studies for regenerating degenerated intervertebral discs by promoting extracellular matrix production and modulating inflammation. However, challenges such as the harsh microenvironment of the disc and limited cell survival post-implantation remain. Recent approaches combining MSCs with hydrogels or extracellular vesicles (EVs) are being explored to enhance cell viability and therapeutic efficacy [33,34,35,36].

Nanotechnology offers advanced implant coatings and drug delivery systems to reduce infection or enhance osteointegration. For instance, nanostructured surfaces on spinal implants can promote better bone–implant integration, while nanoparticle-based delivery systems can provide a controlled release of anti-inflammatory or osteoinductive agents directly at the surgical site. These innovations, though still largely experimental, may complement surgical techniques by addressing tissue-level pathologies and improving long-term outcomes.

Recent studies have demonstrated that nanostructured surfaces on spinal implants can significantly enhance osseointegration. For instance, titanium implants with TiO_2_ nanotube arrays have been shown to improve protein adsorption, promote osteoblast adhesion, and modulate immune responses, thereby facilitating better bone–implant integration [37,38,39].

In addition to surface modifications, nanoparticle-based delivery systems are being explored to provide the controlled release of therapeutic agents at the surgical site. Lactoferrin-anchored, tannylated, mesoporous silica nanoparticles, for example, have demonstrated potential in promoting bone fusion and angiogenesis in spinal fusion models [40].

Furthermore, the use of bioactive nanomaterials, such as metal and metal oxide nanoparticles, has been investigated for their antibacterial and anti-inflammatory properties, which are crucial in preventing postoperative infections and promoting healing [41].

### 3.2. Minimally Invasive Techniques

#### 3.2.1. Endoscopic and Percutaneous Spine Surgery

Fully endoscopic spine surgery has emerged as one of the least invasive surgical options for lumbar disc herniations and spinal canal decompression [42,43]. In endoscopic lumbar discectomy, surgeons use a needle-size tubular endoscope to remove herniated disc fragments through a very small incision [44,45]. Over the past five years, endoscopic techniques have gained popularity worldwide and increasingly in the United States [1]. The proposed benefits include less muscle and soft tissue disruption, reduced blood loss, less postoperative pain, and faster recovery compared to the standard open microdiscectomy, which requires a larger incision and muscle retraction [1,46,47].

Recent evidence suggests that endoscopic discectomy achieves equivalent long-term outcomes to conventional microdiscectomy, while conferring some short-term advantages [43,48,49]. A 2021 systematic review and meta-analysis compared percutaneous transforaminal endoscopic discectomy (PTED) with open microdiscectomy for lumbar disc herniation. It found no significant differences in leg pain relief or functional recovery at 6- to 12-month follow-ups between the two procedures [50]. This aligns with the results of several randomized trials that reported non-inferior pain and ODI improvements with endoscopic discectomy compared to microdiscectomy [43,51,52].

Where endoscopic surgery appears to shine is in perioperative morbidity and recovery time. A large multi-center analysis of the ACS-NSQIP database, from 2015 to 2018, identified over 38,000 single-level lumbar discectomy cases, of which a subset of 175 were endoscopic. Despite the small proportion, the outcomes were telling: patients who underwent endoscopic discectomy had a significantly lower overall complication rate (only 0.6% adverse events) compared to those who had open discectomy (3.4% adverse events). They also had a shorter hospital stay on average (0.8 days vs. 1.15 days, *p* < 0.05) [1]. Although both approaches typically allow for rapid discharge (next-day or even same-day surgery), the endoscopic group’s shorter length of stay suggests some patients were able to go home the same day of surgery, reflecting faster immediate recovery [53]. Operative time in that series was roughly similar between groups [1], dispelling the notion that endoscopic procedures necessarily take longer. These findings indicate that endoscopic techniques can reduce the immediate postoperative pain and risk of complications like surgical site infection or excessive blood loss [51,52,54].

It is worth noting that endoscopic surgery has a learning curve, and outcomes are highly operator dependent [43]. As surgeons have gained experience, reherniation rates and reoperation rates for endoscopic discectomy in recent studies are comparable to open surgery. Some recent RCTs even suggest slightly faster early pain reduction and return to work with endoscopic techniques, although by 1 year the differences equalize [44,47,52]. Overall, the evidence now supports endoscopic lumbar discectomy as a safe and effective alternative to open microdiscectomy for properly selected patients [50]. Key patient factors for success include contained herniations and no severe canal stenosis, as extremely migrated or large fragments may still require an open approach [43,46,51,54].

In addition to discectomy, minimally invasive principles have been extended to lumbar fusions [49,55,56]. Percutaneous pedicle screw fixation involves inserting screws through small stab incisions in the skin, using guidewires and dilation, rather than via an open midline exposure [57,58]. This technique, combined with tubular retractors for performing the decompression and interbody fusion, defines MIS fusion procedures like MIS-TLIF (Transforaminal Lumbar Interbody Fusion) and MIS-PLIF (Posterior Lumbar Interbody Fusion) [42,57]. The advantage of percutaneous instrumentation is that it avoids detaching the paraspinal muscles from the spine; instead, screws are placed with guidance (fluoroscopic or navigation) through muscle planes, and rods are connected subfacially [56,58]. This drastically reduces muscle trauma, bleeding, and postoperative pain [42,47].

High-level evidence from RCTs and meta-analyses in the past five years has solidified the benefits of MIS fusion. A 2020 systematic review and meta-analysis pooled seven RCTs with a total of 496 patients, comparing single-level minimally invasive TLIF to traditional open TLIF [59]. The results showed MIS-TLIF achieved the same therapeutic goals as open TLIF in terms of fusion success and long-term patient outcomes, with significantly less surgical morbidity. Specifically, there was no difference in fusion rates at 12 months (pseudarthrosis incidence ~10% in both and not statistically different) [59] and no difference in long-term leg or back pain scores [59,60]. Functional outcomes were likewise similar, though the MIS group had a marginally better ODI, on average ~3 points lower, *p* = 0.01, which is a small but statistically significant difference favoring MIS [59,60].

MIS-TLIF was associated with reduced intraoperative blood loss, approximately 200 mL less on average, and a shorter hospital stay by roughly two days compared to the open TLIF approach [59,61,62]. Patients undergoing MIS-TLIF typically mobilize earlier and fulfill discharge criteria sooner, primarily due to reduced postoperative pain and lower analgesic requirements [49,56,63]. The main trade-off observed was a modest increase in fluoroscopy time, averaging 48 s more than in open procedures [49,59]. However, the overall operative time was comparable between MIS and open approaches, challenging the notion that MIS techniques are inherently slower. Importantly, complication rates did not differ significantly between the two techniques, indicating no increased risk of perioperative complications with MIS-TLIF [59]. These findings suggest that, when performed with appropriate image guidance, MIS-TLIF does not carry a higher risk of pedicle screw malposition or neurological injury [57]. Table 2 summarizes these comparisons.

A newer frontier that overlaps endoscopy and fusion is endoscopic-assisted interbody fusion (endoscopic TLIF), where an endoscope is used through a tubular retractor to perform discectomy and cage placement with minimal exposure [57]. Early case series in the last five years have shown it is feasible, but evidence is still limited, and it remains technically challenging. It aims to combine the benefits of endoscopic decompression with the stability of fusion for select cases like low-grade spondylolisthesis [51].

#### 3.2.2. Lateral and Oblique Lumbar Interbody Fusion

Minimally invasive anterolateral approaches to the lumbar spine, namely Extreme Lateral Interbody Fusion (XLIF) and Oblique Lumbar Interbody Fusion (OLIF), have gained substantial traction in the last 5–10 years. These techniques allow surgeons to access the lumbar disc space from the retroperitoneal space, avoiding the posterior muscles and bones entirely. By doing so, a larger interbody cage can often be inserted, restoring disc height and lordosis effectively, and with less back muscle disruption. XLIF, introduced in the mid-2000s, uses a direct lateral trans-psoas route, whereas OLIF takes a slightly more oblique anterior-to-psoas trajectory, aiming to avoid traversing the psoas muscle and thus potentially reduce nerve irritation [64].

Both XLIF and OLIF are indicated for lumbar degenerative disease, deformity correction, and spondylolisthesis. A 2023 meta-analysis comparing OLIF to TLIF for degenerative lumbar disease found no significant difference in operative time, complication rates, or fusion rates between the two techniques, confirming their equivalence in achieving surgical goals. However, OLIF demonstrated notable advantages in perioperative outcomes, including significantly lower blood loss, by approximately 150–200 mL, and shorter hospital stays, by 1–2 days, reflective of its muscle-sparing nature [65,66,67].

Importantly, patients undergoing OLIF reported faster early recovery. At 3 months, OLIF was associated with lower back pain scores and better ODI scores compared to TLIF, with a residual ODI benefit (2–3 points) persisting at final follow-up. Leg pain relief was comparable between approaches, as both provide adequate neural decompression [64].

Lateral approaches have also shown superiority in radiographic outcomes. OLIF and XLIF achieved greater improvements in disc height, foraminal height, and segmental lordosis compared to TLIF, making them suitable for sagittal imbalance correction in adult deformity cases [65,67].

While clinical outcomes between XLIF and OLIF are similar for levels L1–L5, their complication profiles differ due to surgical trajectory. A 2022 meta-analysis reported a higher rate of transient postoperative neuropraxia with XLIF (21%) versus OLIF (11%), attributed to the trans-psoas path’s proximity to the lumbar plexus [64]. Conversely, OLIF carries a higher risk of vascular injury (~3.2%) due to its anterior-to-psoas trajectory, often requiring vascular access surgeons, particularly at higher lumbar levels [66,67].

Surgeon expertise and patient selection are key: not every patient is a candidate (XLIF cannot access L5-S1, so OLIF may be needed there, or an anterior ALIF), and careful attention must be paid to avoid each approach’s specific complications. The learning curve can be significant, requiring familiarity with retroperitoneal anatomy [65]. Table 2 highlights how these lateral methods stack up against conventional approaches.

### 3.3. Clinical and Functional Outcomes of New Techniques

A central question for any surgical innovation is the following: Do patients fare better in terms of pain relief, quality of life, and functional recovery? For lumbar spine surgery, patient-centric outcomes include postoperative pain levels, neurological status, disability indices, return to daily activities or work, and the durability of symptom relief. Based on data from the past five years, we can summarize the clinical and functional outcomes associated with the latest advancements.

#### 3.3.1. Postoperative Pain

Patients treated with minimally invasive or technologically guided techniques often experience less early postoperative pain compared to those who underwent traditional open surgery [68]. This is evidenced by lower inpatient pain scores and reduced analgesic requirements in many MIS studies (MIS-TLIF vs. open-TLIF and OLIF vs. MIS-TLIF) [64]. However, by intermediate and long-term follow-up (3, 6, or 12 months), pain outcomes equalize in most comparative studies [5,50]. Essentially, new-tech and conventional groups achieve substantial relief of nerve-related pain if the surgery adequately addresses the pathology [69]. The residual back pain that patients sometimes have from muscle damage or scarring tends to be lower in MIS cohorts, which can slightly tilt patient-reported back pain in favor of MIS even long-term, but differences are usually small [70].

#### 3.3.2. Disability and Functional Recovery

Measured by the ODI or Short From-36 (SF-36) physical component, functional outcomes have likewise been comparable or slightly improved with newer techniques [71]. MIS fusion patients (MIS-TLIF vs. open-TLIF and OLIF vs. MIS-TLIF) in RCTs achieved the same or marginally better ODI improvements at 1 year [59,64]. More striking is the speed of functional recovery: patients often regain mobility faster after MIS procedures. Measures like time to walk unassisted, time to climb stairs, or time to return to work favor MIS [72]. One study on endoscopic vs. open discectomy found patients in the endoscopic group returned to work on average 1–2 weeks sooner. In lumbar fusion, some MIS patients can do light activities in a matter of weeks, whereas open surgery patients might be laid up a bit longer due to wound and muscle healing [60].

#### 3.3.3. Fusion Rates, Radiographic Outcomes, and Infection Rates

The goal of fusion surgery is a solid arthrodesis. It is reassuring that fusion rates have not been compromised by MIS techniques or new technology. High fusion (>90%) is reported at 12 months for both MIS-TLIF and open TLIF alike [59] and similarly high rates for lateral fusion vs. posterior fusion [65]. Use of biologics or adjuncts (BMP, etc.) can affect fusion rates, but those are variables common to both approaches [73]. Radiographically, patients who undergo lateral fusions often have better disc height and alignment, which correlates with possibly better foraminal decompression, and in some studies, this meant better leg pain relief [65], potentially due to indirect decompression. However, generally clinical outcomes like leg pain or neurogenic claudication relief are equivalent if nerves are adequately decompressed by any approach [42].

Recent evidence has consistently shown that minimally invasive spine surgery is associated with lower rates of surgical site infections compared to traditional open procedures. In a 2024 retrospective cohort study involving 150 patients undergoing lumbar decompression, the surgical site infection rate was 5.6% in the open surgery group versus 2.4% in the endoscopic group, suggesting a trend toward improved infection control with MIS techniques, although the difference did not reach statistical significance [74].

Another recent study reported a significantly lower infection rate of 1.5% in the MIS group compared to 3.8% in the open surgery group, reinforcing the clinical benefit of tissue-sparing approaches in reducing postoperative infections [75].

Similarly, a large retrospective analysis of 1442 patients undergoing lumbar spine surgery for degenerative conditions found a markedly lower site infection rate in the MIS group (0.5%) compared to the open surgery group (3.3%), revealing a statistically significant difference (*p* = 0.0003) [76].

These findings support the hypothesis that MIS techniques, by minimizing soft tissue disruption and shortening wound exposure time, can contribute to a lower risk of postoperative infection and improved wound healing.

#### 3.3.4. Recovery Time and Return to Activity

Length of hospital stay (LOS) is a concrete metric reflecting recovery speed. Virtually all comparisons show reduced LOS with MIS techniques: patients go home sooner by roughly 1 day (discectomy) to 2–3 days (fusion) on average [1,59]. This is a patient-centered outcome in that it indicates they have achieved pain control and independent mobility faster [77]. Early mobilization also reduces risks like deep vein thrombosis and atelectasis, indirectly improving outcomes [42]. One randomized trial reported that full-endoscopic lumbar discectomy patients returned to work significantly earlier (median 27 days) than microdiscectomy patients (median 42 days) [1]. For fusions, heavy laborers might be out for 3–6 months regardless of approach, but those with sedentary jobs may resume sooner after MIS [72,78].

#### 3.3.5. Patient Satisfaction and Quality of Life

These subjective measures tend to mirror the objective outcomes. In general, when pain relief is equal and function is equal, satisfaction rates are equal. However, patients do appreciate less invasive surgery (smaller scar and an earlier discharge) and perception of high-tech care can positively influence satisfaction [5,43,77]. Surveys in some studies indicated a higher proportion of MIS patients would choose the same surgery again compared to open patients, although this is not universal [59,79].

#### 3.3.6. Durability and Reoperations

A critical outcome is whether new techniques lead to more reoperations: for example, if an MIS decompression was inadequate and needed revision. Current data show that reoperation rates for recurrent herniation or adjacent level disease did not consistently increase with MIS [80]. Endoscopic discectomy has a similar reherniation risk as microdiscectomy in meta-analysis. In the Spine database study, 3-year reoperation was statistically identical between robotic and non-robotic fusions (~5–6%) [8], indicating the technology did not reduce or increase the need for later surgery [81]. Fusion constructs placed by MIS have held up similarly to open constructs in mid-term follow-ups. Thus, the long-term durability of outcomes appears to be maintained with new techniques [2].

#### 3.3.7. Learning Curve of Minimally Invasive Spine Surgery

These techniques are associated with a notable learning curve. Surgeons transitioning from open to MIS approaches often face technical challenges, including limited working space, altered anatomic visualization, and a greater reliance on fluoroscopy or navigation for orientation. Systematic reviews by Yong et al. and Kong et al. found that proficiency in MIS-TLIF typically requires 20 to 40 cases, with operative times, complication rates, and conversion to open surgery decreasing as experience increases [82,83].

Furthermore, studies show that, early in the learning curve, MIS procedures are associated with longer operative durations and a higher rate of technical complications such as dural tears or suboptimal implant placement [83]. This underscores the importance of structured training, mentorship, and simulation-based practice for safe adoption. The integration of technologies like robotics, navigation, and augmented reality can help mitigate the steepness of the learning curve by enhancing intraoperative visualization and guidance, though these systems themselves also require training [84].

Similarly, robotic-assisted spine surgery follows a distinct learning curve. Early robotic procedures are often characterized by longer setup and registration times (30–40 min additional in the first 10–15 cases), increased intraoperative troubleshooting, and initially prolonged operative durations. A systematic review by Pennington et al. found that proficiency in robotic spine surgery is typically achieved after 20 to 30 cases, with subsequent improvements in workflow integration, setup efficiency, and reduction in technical errors [85]. Importantly, studies report that complication rates remain low even during the early learning phase, suggesting that robotic platforms maintain safety while the surgical team gains experience.

## 4. Discussion

This review highlights the clinical implications of recent advancements in lumbar spine surgery, emphasizing both their benefits and limitations. Over the past five years, the adoption of robotic systems, computer navigation with augmented reality, and minimally invasive techniques has reshaped surgical practice, refined procedural accuracy, and improved perioperative outcomes without compromising the fundamental goals of pain relief and neural decompression.

One of the most significant contributions of new technologies is the improvement in surgical precision and reproducibility. Robotic systems allow for highly accurate pedicle screw placement, minimizing the risk of misplacement and potential neurological complications [6]. This level of precision is particularly advantageous in complex cases, such as deformity corrections and revision surgeries, where anatomical distortions challenge freehand techniques [5,28]. Similarly, augmented reality navigation enhances intraoperative visualization by integrating digital overlays within the surgeon’s field of view, improving workflow efficiency and reducing reliance on traditional fluoroscopic guidance [15,21]. Artificial intelligence, though still in its early stages, offers promising applications in surgical planning, intraoperative guidance, and predictive analytics, potentially aiding in personalized surgical decision-making [26,86]. Meanwhile, minimally invasive approaches, particularly endoscopic and percutaneous techniques, continue to gain traction due to their ability to reduce muscle trauma, decrease postoperative pain, and shorten recovery times [1]. These innovations align with the principles of enhanced recovery after surgery, facilitating earlier mobilization and discharge while minimizing overall physiological stress [42].

Despite these advantages, challenges remain in integrating these technologies into routine practice. The learning curve associated with robotic navigation and endoscopic techniques is substantial, often leading to increased operative times and a higher risk of technical errors during the initial phase of adoption [48]. Adequate training programs, including simulation-based learning, are necessary to mitigate these challenges and ensure safe implementation. To address the learning curve associated with advanced spine surgery techniques, it is crucial to emphasize the role of structured university-based educational programs. These initiatives, such as cadaver labs, hands-on fellowships, and interdisciplinary research courses, provide comprehensive and unbiased training environments. Academic curricula ensure that future surgeons acquire both the technical and critical-thinking skills needed for the safe and effective adoption of emerging technologies.

Another concern is the potential over-reliance on technology, which can introduce new failure points, such as navigation errors, system malfunctions, or robotic calibration issues [5,12]. Surgeons must maintain fundamental technical skills and be prepared to revert to conventional methods when necessary, emphasizing that technology should augment, rather than replace, clinical judgment. Additionally, the economic impact of these innovations must be carefully evaluated [11]. High upfront costs, ongoing maintenance, and disposable instrumentation contribute to significant financial burdens, particularly for smaller institutions or resource-limited settings [87]. While preliminary data suggest that robotic and navigated spine surgeries may reduce complication rates and revision procedures, thereby offsetting some costs, definitive cost-effectiveness studies are still lacking [12].

A critical and often debated topic is the correlation between decompression surgery and the indication for spinal fusion. While decompression alone may be sufficient for isolated neural compression, many clinical scenarios like segmental instability, degenerative spondylolisthesis, or recurrent stenosis necessitate the addition of fusion to prevent progression or recurrence [88,89]. The decision-making process remains nuanced and patient-specific, influenced by both radiographic findings and dynamic biomechanical considerations [90,91]. Future studies are warranted to better stratify which patient populations derive the greatest benefit from adjunctive fusion following decompression.

One of the key limitations of the current body of evidence is the relatively short follow-up duration in most studies, often limited to one or two years. Long-term data are needed to determine whether the advantages of robotic and minimally invasive techniques translate into the superior durability of outcomes, such as reduced adjacent segment degeneration or lower rates of chronic postoperative pain [81,92]. Similarly, while early infection rates appear lower with minimally invasive surgery, the impact on long-term implant stability and late-onset complications requires further study [59]. The quality of available evidence also varies depending on the technology in question. Robust Level I evidence from randomized controlled trials supports many aspects of minimally invasive fusion and endoscopic discectomy, while data on augmented reality and artificial intelligence remain limited to feasibility studies and small cohort analyses [93]. Although robotic-assisted surgery has been evaluated in meta-analyses, many studies are industry-sponsored or non-blinded, raising concerns about potential bias.

Future research should focus on refining patient selection criteria for these advanced techniques, identifying subgroups who may derive the greatest benefit from minimally invasive or technology-assisted approaches. For instance, elderly and frail patients may experience significant advantages from muscle-sparing procedures, whereas complex deformity cases may still require open surgical exposure despite technological aids. As these innovations become more widespread, they must be integrated into surgical training programs to ensure that future generations of spine surgeons are proficient in both traditional and modern techniques. Additionally, long-term comparative studies examining clinical and functional outcomes beyond the immediate postoperative period will be crucial in determining the true value of these advancements. The convergence of robotics, navigation, artificial intelligence, and augmented reality into comprehensive surgical platforms represents the next frontier in spine surgery, potentially enabling semi-autonomous procedures and data-driven intraoperative decision-making.

## 5. Conclusions

The latest advancements in lumbar spine surgery have proven to be evolutionary rather than revolutionary. They build upon the solid foundation of conventional techniques, making surgery more precise, less invasive, and potentially safer. The relevance for clinical practice is significant: spine surgeons now have an expanded toolkit to tailor the best approach for each patient, which is at the heart of personalized surgical care. By integrating robotics, navigation/AR, and MIS techniques, surgeons can achieve outcomes that meet or exceed those of the past, often with fewer patient risks and faster recoveries. As the field progresses, a continued commitment to the evidence-based adoption of innovations will ensure that these advancements truly benefit patients and advance the standard of care in lumbar spine surgery.

## Figures and Tables

**Table 1:** Key advantages and current evidence of robotic-assisted, intraoperative navigation, and AR and AI for lumbar spine surgery.

Technology	Key Advantages	Current Evidence (Last 5 Years)
Robotic-Assisted Surgery	High precision in pedicle screw placement (≈92–94% accuracy vs. ~70% freehand).	Accuracy: Significantly higher Grade A screw placement vs. conventional freehand.
	Reduced intraoperative blood loss and radiation exposure in some studies.	Perioperative: Less blood loss and shorter hospital stays observed in RCT meta-analysis. No increase in overall OR time.
	Facilitates minimally invasive approaches (percutaneous instrumentation).	Outcomes: No improvement in 90-day or long-term clinical outcomes versus non-robotic surgery in matched comparisons. Similar pain relief and fusion rates.
		Limitations: High initial cost; learning curve; few studies on cost-benefit (one analysis shows cost-effectiveness if revisions are reduced).
Intraoperative Navigation (Conventional 3D navigation systems)	Improves screw placement accuracy over fluoroscopy alone.	Accuracy: Established improvement in pedicle screw accuracy vs. freehand.
	Avoids reliance on surgeon’s anatomic estimates, enhancing safety in complex anatomy.	Workflow: Can increase setup time and requires surgeon to look away to a separate screen, which may disrupt workflow.
		Outcomes: Associated with reduced screw misplacement-related complications, but direct impact on long-term outcomes is unclear. Widely adopted especially in deformity and revision cases.
Augmented Reality Navigation	Projects navigation cues into surgeon’s field of view (via headsets or displays) resulting in improved ergonomics (surgeon keeps eyes on patient).	Accuracy: Non-inferior to standard navigation; AR-guided pedicle screw placement accuracy comparable to conventional navigation and far better than freehand. Early head-mounted AR studies report accuracy on par with navigation (differences not statistically significant).
	Workflow efficiency: Easier, more intuitive instrument guidance, potentially shorter learning curve for navigation.	Workflow: Qualitatively improved; surgeons report greater ease and faster confirmation of trajectories. AR deemed a “meaningful addition” to traditional methods.
	May reduce need for fluoroscopy (lower radiation) by providing continuous visual guidance.	Outcomes: No clinical outcome or cost data yet. Studies so far are small; need prospective trials to confirm any reduction in operative time or complication rates.
Artificial Intelligence (AI) (machine learning for planning, guidance, and decision support)	Preoperative: Automates image analysis and surgical planning (3D reconstructions and optimal screw trajectories).	Feasibility: AI-driven tools have been implemented in pilot studies with satisfactory accuracy and safety profiles (e.g., AI planning yielding proper screw placement and AI-assisted imaging matching expert readings).
	Intraoperative: Enhances robotics and navigation (e.g., adaptive trajectory adjustments and real-time tissue recognition).	Radiation/Precision: AI-assisted navigation shown to reduce reliance on fluoroscopy and maintain or improve accuracy versus standard techniques.
	Postoperative: Predicts outcomes and complication risks (personalized prognostics).	Clinical impact: No proven superiority in patient outcomes yet. Lacks RCTs; current evidence (11 studies review) shows no clear outcome advantage of AI assistance, although processes are improved.
	Can integrate with AR/VR to improve simulation and training.	Future: Rapidly evolving; expected to enhance surgeon decision-making and possibly enable semi-autonomous surgeries.

**Table 2:** Comparison of the key findings from the sources (2019–2024).

Procedures	Surgical Outcome Measures	Key Findings
Endoscopic Discectomy vs. Open Microdiscectomy	Long-term leg pain relief and functional improvement	No significant difference. Both approaches yield comparable outcomes for sciatica at 6–12 months.
	Hospital Stay	Endoscopic shorter (e.g., ~0.8 vs. 1.1 days) with more same day discharges.
	Complications	Endoscopic has lower risk of adverse events (0.6% vs. 3.4% in one large series), including lower infection rates.
	Recovery	Faster early mobility and less postoperative pain medication reported with endoscopic technique in several studies (due to minimal muscle disruption).
MIS TLIF vs. Open TLIF (single-level fusion)	Perioperative	Less blood loss with MIS (~200 mL reduction).
		Shorter hospitalization by ~2 days (earlier ambulation and discharge).
		Fluoroscopy use is higher in MIS (by ~48 s) due to percutaneous screw placement.
	Complications	No significant difference in overall complication rates. MIS approach does not increase neurological or hardware-related complications when performed with navigation/experience.
	Fusion Rate	Equivalent between groups (~90% at 12 months). MIS does not compromise bony fusion healing.
	Clinical Outcomes	Similar pain scores at 1 year (both groups improve greatly). ODI slightly better in MIS group (by ~3 points), indicating a small functional benefit. Long-term outcomes (up to 5 years) show no differences in pain/disability trajectories.
Lateral/Oblique Fusion (XLIF/OLIF) vs. Posterior Fusion (TLIF)	Operative Time	No significant difference (lateral approaches as efficient as TLIF for single level).
	Blood Loss	Lower in lateral (XLIF/OLIF)—often by 150–250 mL less than TLIF.
	Hospital Stay	Shorter with XLIF/OLIF (mean 1–2 days shorter) due to reduced pain and quicker mobilization.
	Pain and Disability	Faster early improvement in OLIF vs. TLIF. Lower VAS back pain at 3 months and better ODI at 3 months and final follow-up (2–3 point advantage). Leg pain relief similar between groups.
	Fusion and Alignment	Fusion rates high and comparable. XLIF/OLIF yield greater disc height and foraminal height restoration, aiding indirect decompression. Similar or slightly better segmental lordosis gain vs. TLIF, depending on levels fused.
	Complications	Overall rates similar, but profile differs. OLIF: risk of vascular injury (~3%) higher than TLIF (near zero). XLIF: risk of lumbar plexus neuropraxia (transient thigh numbness/weakness) ~10–20% vs. much lower in TLIF. No significant difference in infection rates (both are low due to minimal incisions).

## Data Availability

Not applicable.

## Abbreviations

The following abbreviations are used in this manuscript:

LSS	Lumbar Spine Surgery
AI	Artificial Intelligence
AR	Augmented Reality
MIS	Minimally Invasive Surgery
RCT	Randomized Controlled Trial
XLIF	Extreme Lateral Interbody Fusion
OLIF	Oblique Lateral Interbody Fusion
AMSTAR	Assessing the Methodological of Systematic Reviews
3D	Three Dimensions
CT	Computer Tomography
OP	Operative Room
ODI	Oswestry Disability Index
PTED	Percutaneous Transforaminal Endoscopic Discectomy
TLIF	Transforaminal Lumbar Interbody Fusion
PLIF	Posterior Lumbar Interbody Fusion
ALIF	Anterior Lumbar Interbody Fusion
SF-36	Short Form 36
LOS	Length of Hospital Stay
